# A new deep learning-based technique for rice pest detection using remote sensing

**DOI:** 10.7717/peerj-cs.1167

**Published:** 2023-03-06

**Authors:** Syeda Iqra Hassan, Muhammad Mansoor Alam, Usman Illahi, Mazliham Mohd Suud

**Affiliations:** 1 Universiti Kuala Lumpur British Malaysian Institute, Kuala Lumpur, Malaysia; 2 Department of Electrical Engineering, Ziauddin University, Karachi, Pakistan; 3 Faculty of Computing, Riphah International University, Islamabad, Pakistan; 4 Malaysian Institute of Information Technology, University of Kuala Lumpur, Kuala Lumpur, Malaysia; 5 Faculty of Engineering and Information Technology, University of Technology Sydney, Sydney, Australia; 6 Faculty of Computing and Informatics, Multimedia University, Cyberjaya, Selangor, Malaysia; 7 Electrical Engineering Department, Faculty of Engineering and Technology, Gomal University Dera Ismail Khan, Dera Ismail Khan, Pakistan

**Keywords:** Remote sensing, Deep learning, Smart agriculture, Rice production, Stem Borer, Hispa

## Abstract

**Background:**

Agriculture plays a vital role in the country’s economy and human society. Rice production is mainly focused on financial improvements as it is demanding worldwide. Protecting the rice field from pests during seedling and after production is becoming a challenging research problem. Identifying the pest at the right time is crucial so that the measures to prevent rice crops from pests can be taken by considering its stage. In this article, a new deep learning-based pest detection model is proposed. The proposed system can detect two types of rice pests (stem borer and Hispa) using an unmanned aerial vehicle (UAV).

**Methodology:**

The image is captured in real time by a camera mounted on the UAV and then processed by filtering, labeling, and segmentation-based technique of color thresholding to convert the image into greyscale for extracting the region of interest. This article provides a rice pests dataset and a comparative analysis of existing pre-trained models. The proposed approach YO-CNN recommended in this study considers the results of the previous model because a smaller network was regarded to be better than a bigger one. Using additional layers has the advantage of preventing memorization, and it provides more precise results than existing techniques.

**Results:**

The main contribution of the research is implementing a new modified deep learning model named Yolo-convolution neural network (YO-CNN) to obtain a precise output of up to 0.980 accuracies. It can be used to reduce rice wastage during production by monitoring the pests regularly. This technique can be used further for target spraying that saves applicators (fertilizer water and pesticide) and reduces the adverse effect of improper use of applicators on the environment and human beings.

## Introduction

Pakistan is considered a major rice producer in rice-producing and exporting countries. The Islamic Republic of Pakistan ranked 10th number producing 7 million tons of rice each year, and it contributes to 1.3% of rice exports in the international market, but the production rate is reducing each year due to late prediction of problems arising in crops (Abbas, 2022). Rice production controls the complete production and consumption of crops worldwide. In Pakistan, rice is considered the leading food, and its yield is about 2,70.6 tons of rice has been permitted for commercialism (Memon, 2013). The use of technology in agriculture plays a vital role in making farming precise.

Precision agriculture plays a significant part in agricultural areas for sustainability (Bongiovanni & Lowenberg-Deboer, 2004; Hunt & Daughtry, 2018), and since the 1980s, remote sensing has become an important element of these efforts (Mulla, 2013). Remote sensing is very important in the management of soil (Ali et al., 2015; Carrão et al., 2016), management of pests (Kelly & Guo, 2007; Acharya & Thapa, 2015), detection of weeds (Peña et al., 2013; Thorp & Tian, 2004; Lamb & Brown, 2001; López-Granados & Jurado-Expósito, 2006), surveying (Roslimz et al., 2021), monitoring (Fernandez-Carrillo et al., 2020) vegetation health, and vigor (Berni et al., 2009). Technology and agriculture are some of the best combinations that give financial benefits and improve the economy.

**Figure 1:**
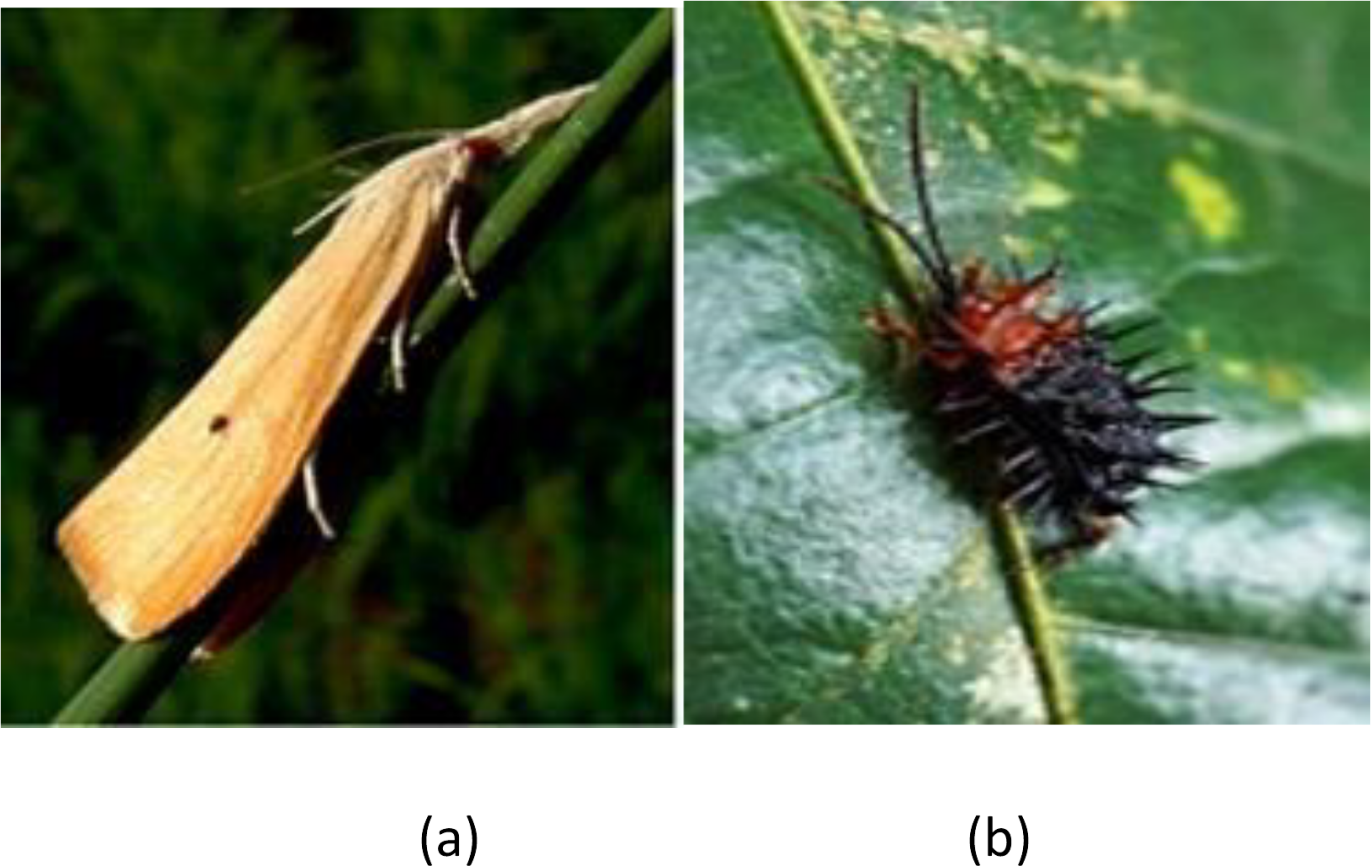
Rice pets: (A) stem borer; (B) Hipsa.

In the financial year 2019 alone, there was a reduction of 3.3; this is primarily because of the stem borer and Hispa in the rice crops, which are the reason for the massive damage. Figure 1 displays the sorts of common pests in rice crops, including stem borer and its categories and Hispa. The Hispa inflicted extreme yield losses on the crop yearly, and stem borers frequently damaged crops from the seedling stage to maturity (Islam, 1975). Stem borer attacks at different stages and leaves its attack symptoms. In any case, caused by the larvae that bore the canes to feed themselves with the corresponding inner tissues. Stem borer includes different categories of attack in the circular path, affecting the rice yield by >70% (Rubia et al., 1989).

Hispa is an insect that is about 5 mm in measurement. Its foods by rasping the leaf surface, which results in the appearance of white lines on the leaf’s surface. The female Hispa lays eggs in these rubbings, and the growth of this pest lasts to increase at a speedy rate. If it is not recognized in time, by postponing the use of pesticides, Hispa can attack and eliminate fields of rice crops within three weeks (Elkhalfia, 2021). Rice Hispa plights the side of leaf edges’ leaving only the lower shell. It conjointly underpasses through the leaf tissues, and plants decrease dynamically, Once the damage is severe. The unwanted plants (weeds) in and near rice crops are grown by the farmer as a substitute to avoid damage from pests. The severe damage mainly occurs on pre-moon soon or initially during the rainy season and leaves the precipitation, most minor daytime-nighttime temperature differential for a variety of days, and a high RH square measure promotes the insect’s growth. Hispa usually attacks in rainfed and irrigated land environments and spread quickly in vegetative periods of rice during the summer season, which causes a loss of 35–65%, and a loss of 100% occurs when transplanted after the flood (Burhan et al., 2020).

Monitoring these pests and identifying the infected location using remote sensing is introduced, which helps to precisely monitor and identify the infected location. The data or images captured by remote sensing are geolocated and georeferenced, which helps highlight the infected site so that desired operations can perform over there. The remote sensing of rice-growing areas not only contributes to the precise mapping of rice areas and the assessment of the dynamics in rice-growing regions but also can contribute to reap prediction modeling. Moreover, the analyses of plant diseases, the assessment of rice-based gas (methane) emission because of vegetation submersion, the investigation of erosion-control-adapted agricultural systems, and the assessment of system services in rice-growing areas can be handled (Kuenzer & Knauer, 2013).

UAVs is developed early in the last century, but in the last decade, drowns are rapidly growing in different sizes, shapes, capacities, and capabilities (Colomina & Molina, 2014). UAVs are widely used in precision agriculture (Shahbazi, Theau & Menard, 2014; Shao, 2012; Roslimher crop et al., 2021). In current periods, remote sensing techniques are gaining much attention, which helps in gaining useful information regarding forest and agriculture management and also helps in increasing the volume and growth of farms (Lyons, 1966).

In the recent decade, with the expansion of sensors, computers, and computational techniques, the pertinence of remote sensing in agriculture used to extract aerial data (Asner et al., 2005) and satellite imagery data (Thakur, Swamy & Nain, 2014), which leads to estimating volume and different calculations of the forest, agriculture indexes, and also the unusual changings for crops results in pests, and diseases (Lu, 2006; Koch, 2010; Singh, 2017).

Pests (rice stem borer (RSB) and Hispa) are the yearly source of substantial financial losses. Control efforts rely heavily on chemical insecticides, which leads to severe problems such as insecticide resistance, environmental pollution, and food safety issues. Therefore, developing alternative pest control methods is an important task. Therefore, this research was intended to evaluate the yield production loss on rice due to stem borer and Hispa infestation, which may support the development of a productive strategy for managing the significant pests of rice.

For the above-stated purpose, an automatic intelligent system is required to identify and classify pests. The use of remote sensing and algorithms helps manage agricultural land free from pests and can also control at a very early stage. Remote sensing delivers the crucial methodology and technology to monitor, map, and observe rice-growing ecologies over huge areas, at frequent time intervals, to understand rice-growing areas under a diversity of features. AI algorithms along with remote sensing contribute a lot in many fields and now their use in the agriculture area is becoming popular. Authors in Zhang et al. (2019) planned spectral collections to support the classification of cultivated land area, in Guo et al. (2022), monitoring and identifying the nutritional status and condition of growth in maize crops. Authors in Biffi et al. (2021) detect apple fruit using deep learning techniques.

The use of algorithms like Support Vector Machine (SVM), K-mean clustering, and other algorithms related to image processing can extract features manually but has a lot of limitations (Phadikar, 2008). These techniques may only work well to detect images within the training data set and fail when introduced to images outside the trained dataset (Mahle, 2016). Deep learning is gaining attention for the detection of pests (Mahle, 2016; Pethybridge & Nelson, 2015; Stewart & McDonald, 2014; Bock et al., 2009; Bai et al., 2017), Pests in cucumbers (Zhang et al., 2017a), and pests in citrus trees (Dorj, Lee & Yun, 2017) additionally feature extraction and image segmentation of crop pests and diseases are recognized and detected by many algorithms (Zhang et al., 2017b; Du et al., 2016; Atole, 2018; Ren et al., 2016). So, deep learning algorithms and remote sensing are being conducted for pest detection in rice crops. In this article, the comparison of deep learning models is provided to ease the selection of the efficient technique for further proceedings.

To summarize, the contributions in this article are: (1) we build a data set for rice pests; (2) we provide a comparative analysis of algorithms to identify the efficient algorithm, which further helped us to achieve and modify the algorithm for achieving precise output; (3) The modified YO-CNN; (4) The set of image detection metrics is proposed, which is based on deep learning classifier.

## Materials & Methods

**Figure 2:**
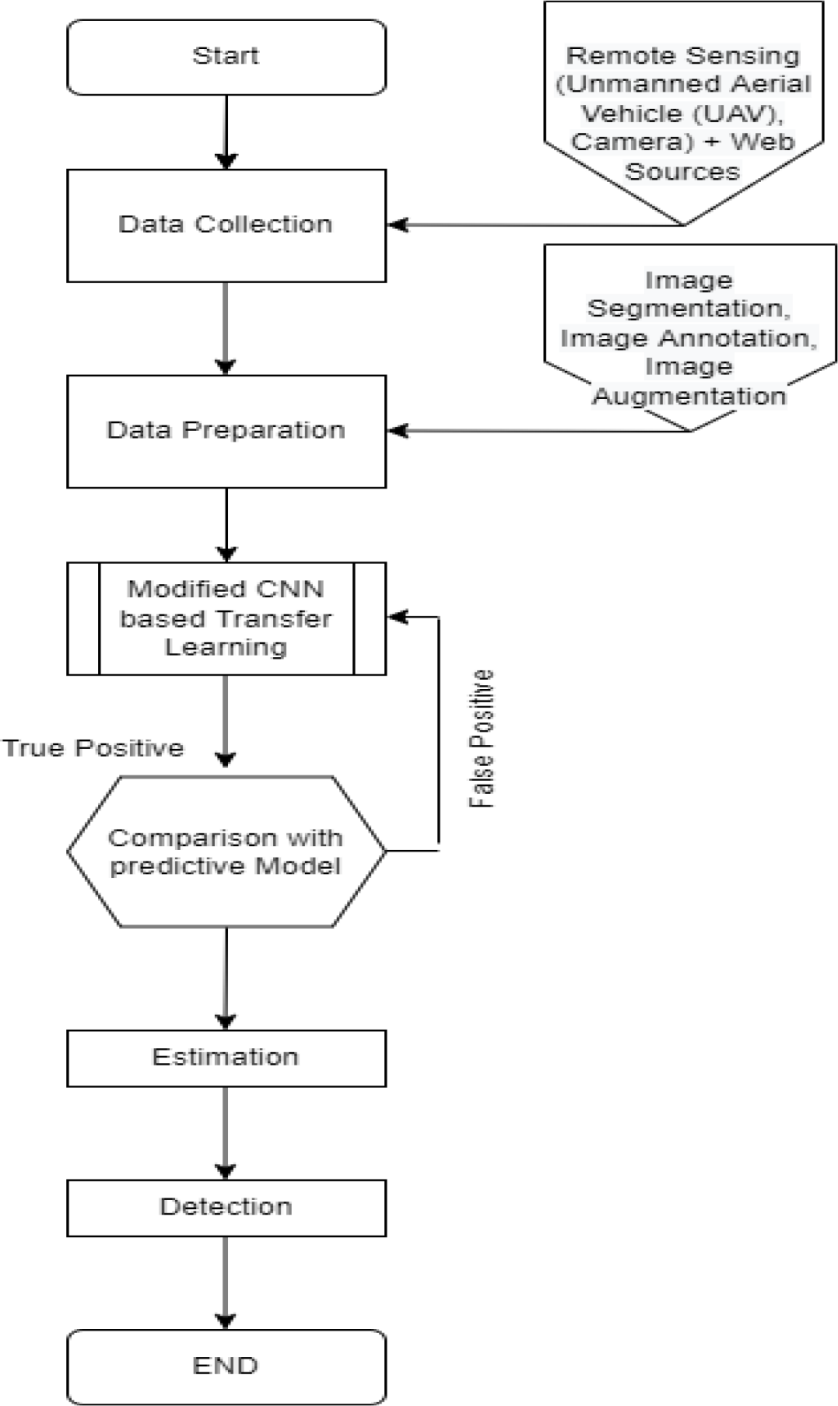
Flow diagram of methodology.

This section provides the methodology for the rice pest detection technique and is further divided into three sub-sections, including (a) image datasets, (b) data preprocessing, and (c) data/image augmentation. An overview of the methodology used in the research is presented in Fig. 2. Data is collected from UAVs, and external sources are prepared for training to detect pests from rice crops. The image is segmented, augmented, and annotated before training. Firstly, we trained data on pre-trained models and analyzed their results by comparing the accuracy of the pre-trained model, leading us towards implementing a modified CNN-based model, which provides a more precise detection than other models with 0.980 accuracies.

### Datasets description

**Figure 3:**
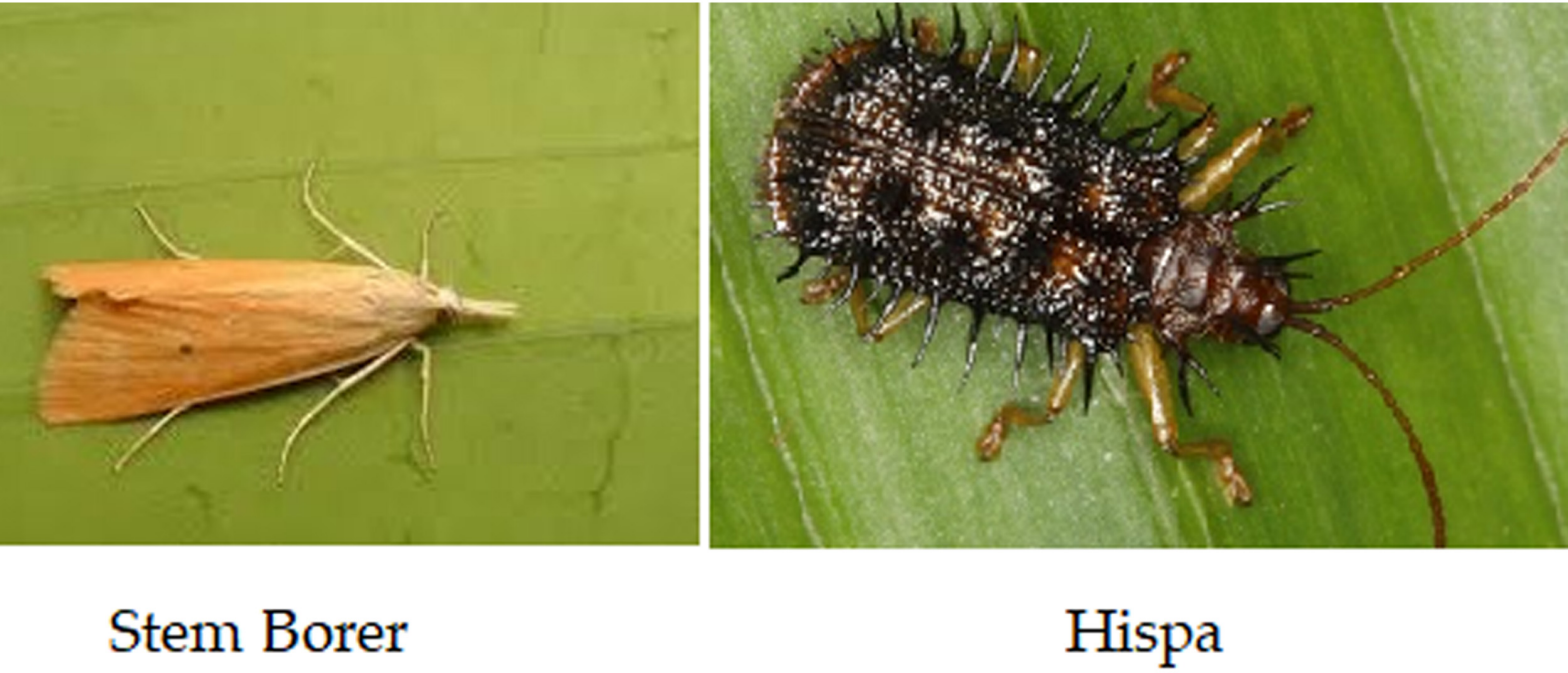
Data collection using UAV.

The dataset collected during the research comprises images of rice pest lesions, collected in Tando Muhammad Khan, Sindh, Pakistan, between June and August 2021 (Khan, 2023). The dataset consists of rice plants infected by rice Stem borer (Islam, 1975) and Hispa (Burhan et al., 2020). These images are captured by the UAV DJI Phantom 4 Pro with a predefined flight path see Fig. 3. The UAV flew autonomously over the rice field at a height of 35 m in an area of 40 acres. The image collection is focused on occurrences of infestation.

Using UAV technology for monitoring and identifying pests in rice crops is an economical solution in terms of time and cost as well as less human effort with minimized errors. The specifications of the UAV in the research for aerial inspection are presented in Table 1.

**Figure 4:**
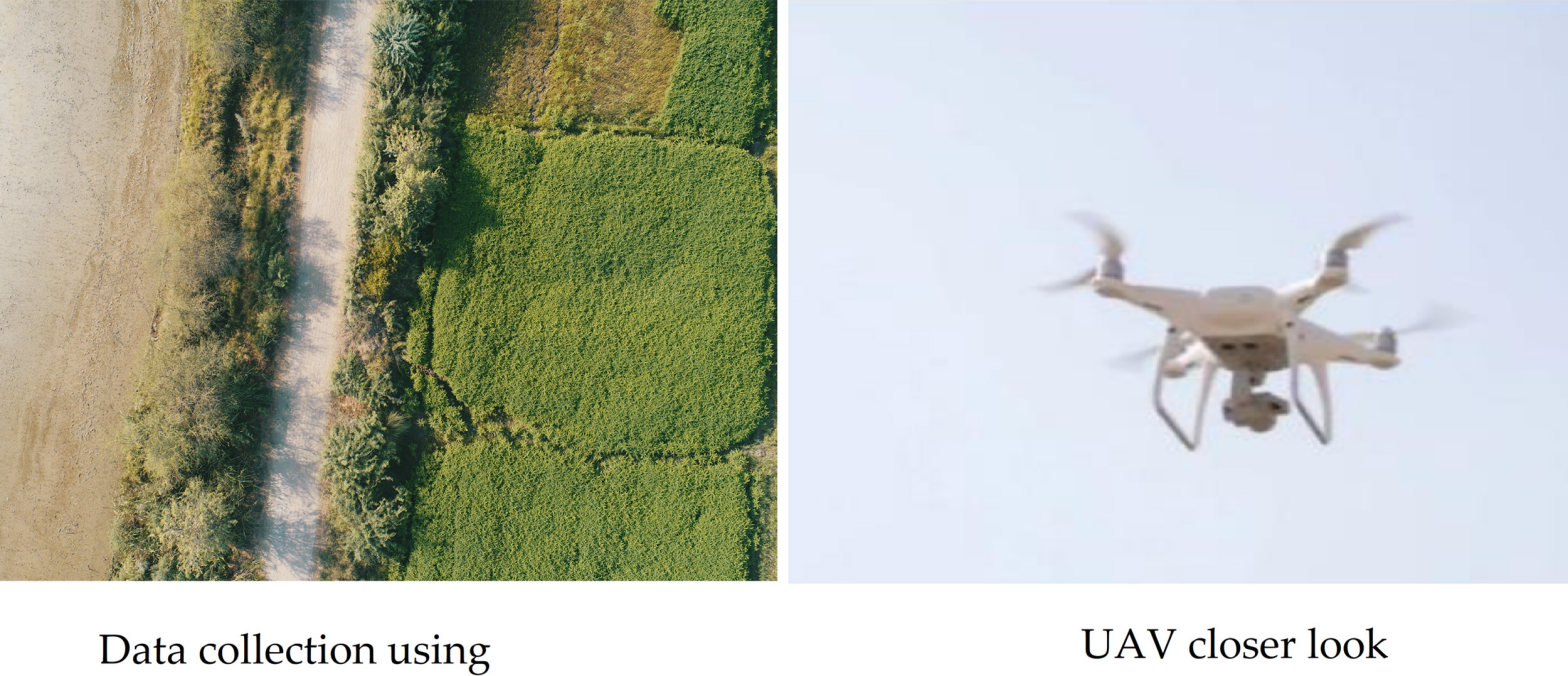
Data set with clear features: (A) stem borer; (B) Hispa.

The dataset comprises external datasets and real images of Stem Borer and Hispa (Roul, 2021; Kaggle, 2022), which shows the precise features of pests as shown in Fig. 4.

The collection of datasets continues for about two days. The dataset includes ground reality and external sources (Roul, 2021; Kaggle, 2022). The farmers know the local name of pests, so the first issue is the language barrier as the farmers do not know the English name of the pests. They use local names or terms for pests. So, the involvement of a specialist in this field or agronomist helped a lot to communicate with the farmers and assign a tag or label the images by indicating the names of pests. The details of the data set, which consists of training and testing data sets, are provided below in Table 2. The data set is divided based on the number of pests in the images. 2,600 are for training which is 80% of the data, and 400 images are considered for testing images which is almost 20% of the data while considering pests in the image.

**Table 1:** UAV specifications for collecting data.

**Parameters**	**Specification**
Weight	1,388 g
Battery Capacity	5,870 mAh
Battery Type	LiPo 4S
Flying altitude	35 m
Speed	3.4 m/s
Missions time	83 min and 37 s
Total No. of flights	8
Camera	1” CMOS Effective pixels:
Mechanical Shutter Speed	8 - 1/2,000 s
Electronic Shutter Speed	8 - 1/8,000 s
Image types	JPEG, DNG (RAW), JPEG
Key points	96,988,666
Tie points	3,996,109

**Table 2:** Statistical imagery data for training and testing the models.

**Dataset of Rice Pests (RSB) and (H)**	**No. of images**
Images used in training	2,600
Images used in testing	400
Total	3,000

**Figure 5:**
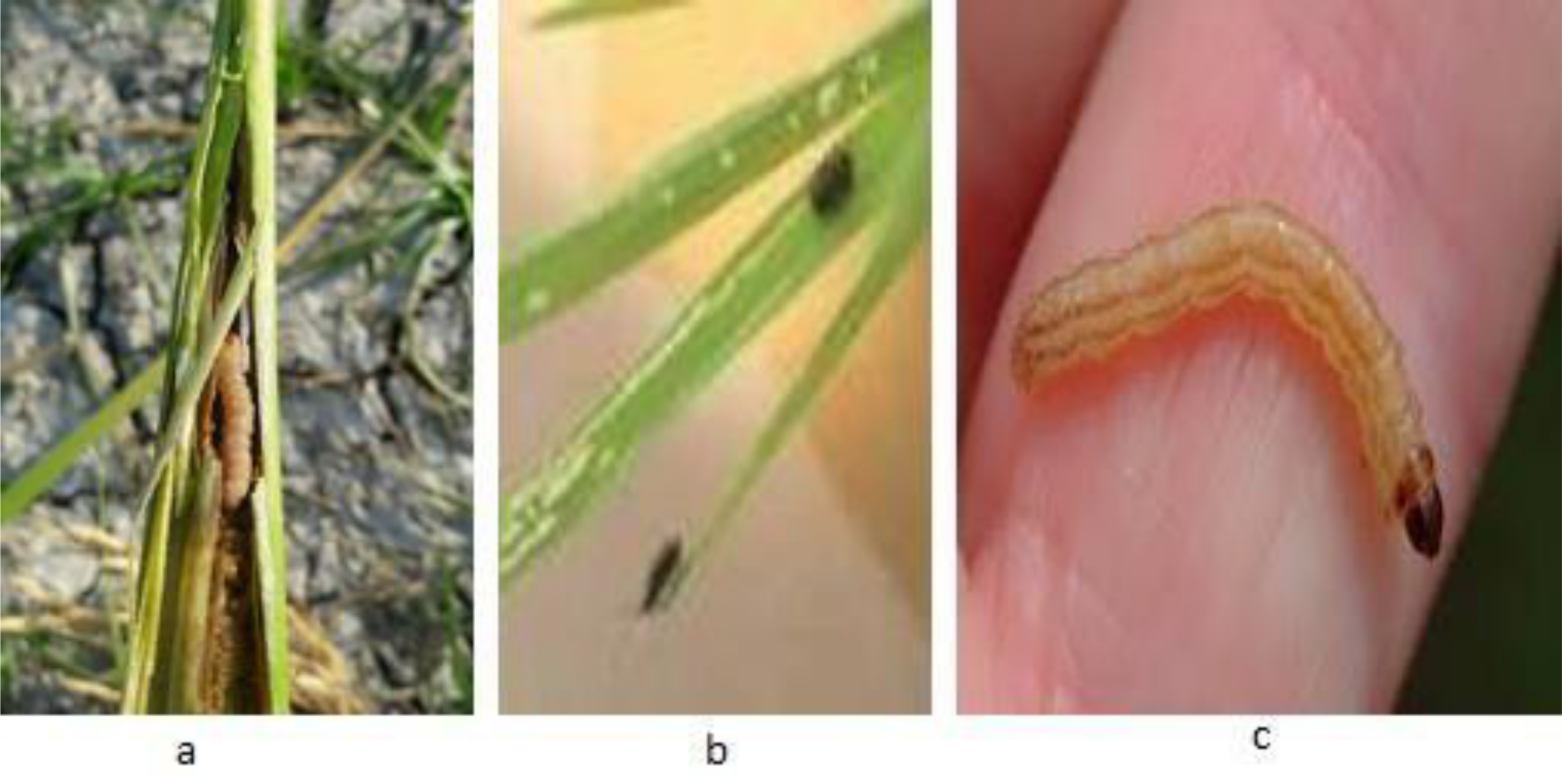
Pests with the original background: (A) stem borer, (B) Hispa, (c) stem borer.

Since the drone imageries were taken from natural rice crop fields, a variation of surroundings can be visualized in the images. Some images consist of more than one leaf; some have soil, and also fingers can be pictured in some images shown in Fig. 5. The collected data are in raw form, and training requires pre-processing. So, in the following sub-section, the pre-processing procedure is explained in detail.

### Data pre-processing

**Figure 6:**
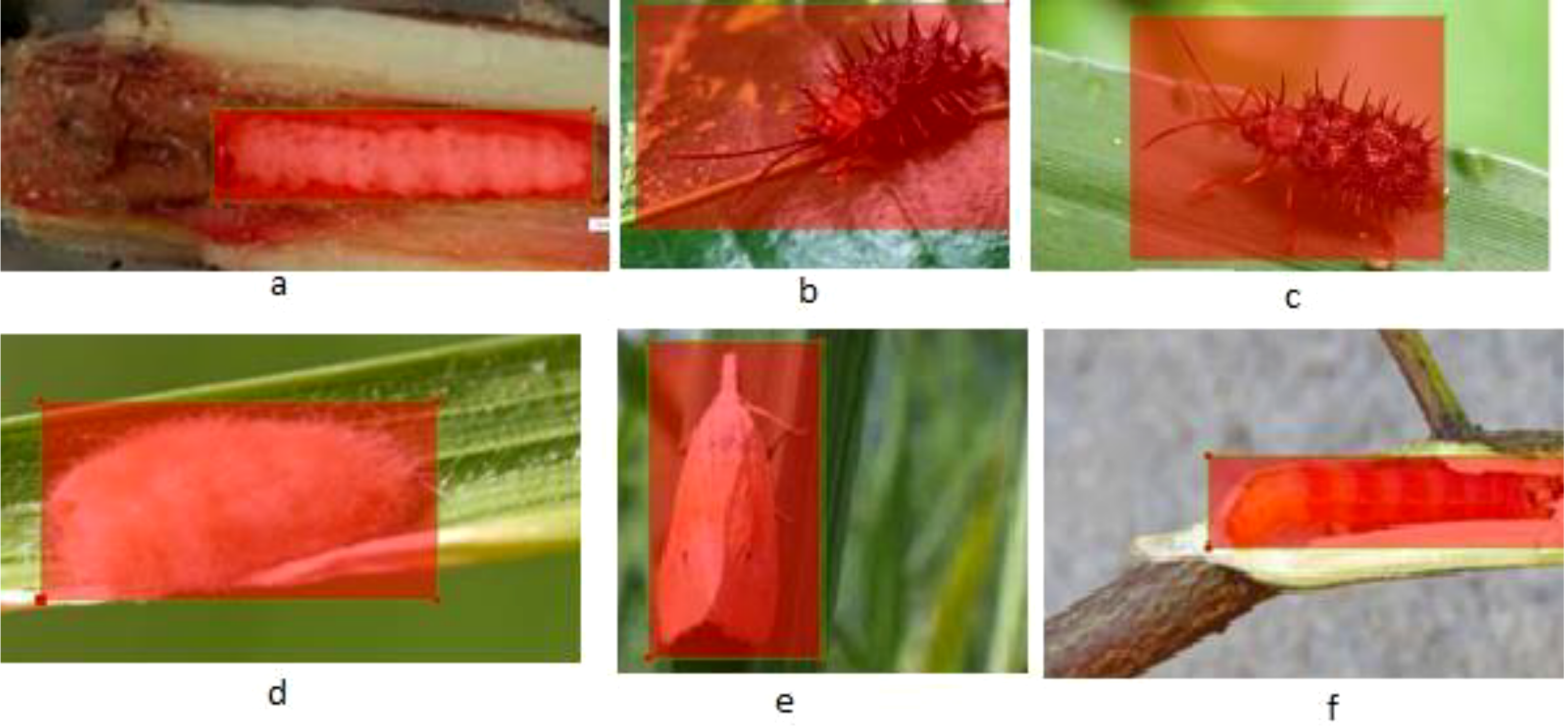
Annotation area selected in the red box for training.

The annotation area of the rice stem borer and Hispa are shown in Fig. 6. The annotation includes clear images that provide a clear vision of the features of pests so that the model will be highly efficient in accurately recognizing pest types.

 After annotation, the image processing technique is applied for the segmentation of the leaf and also for the removal of shadow and background to specify the interested area.

**Figure 7:**
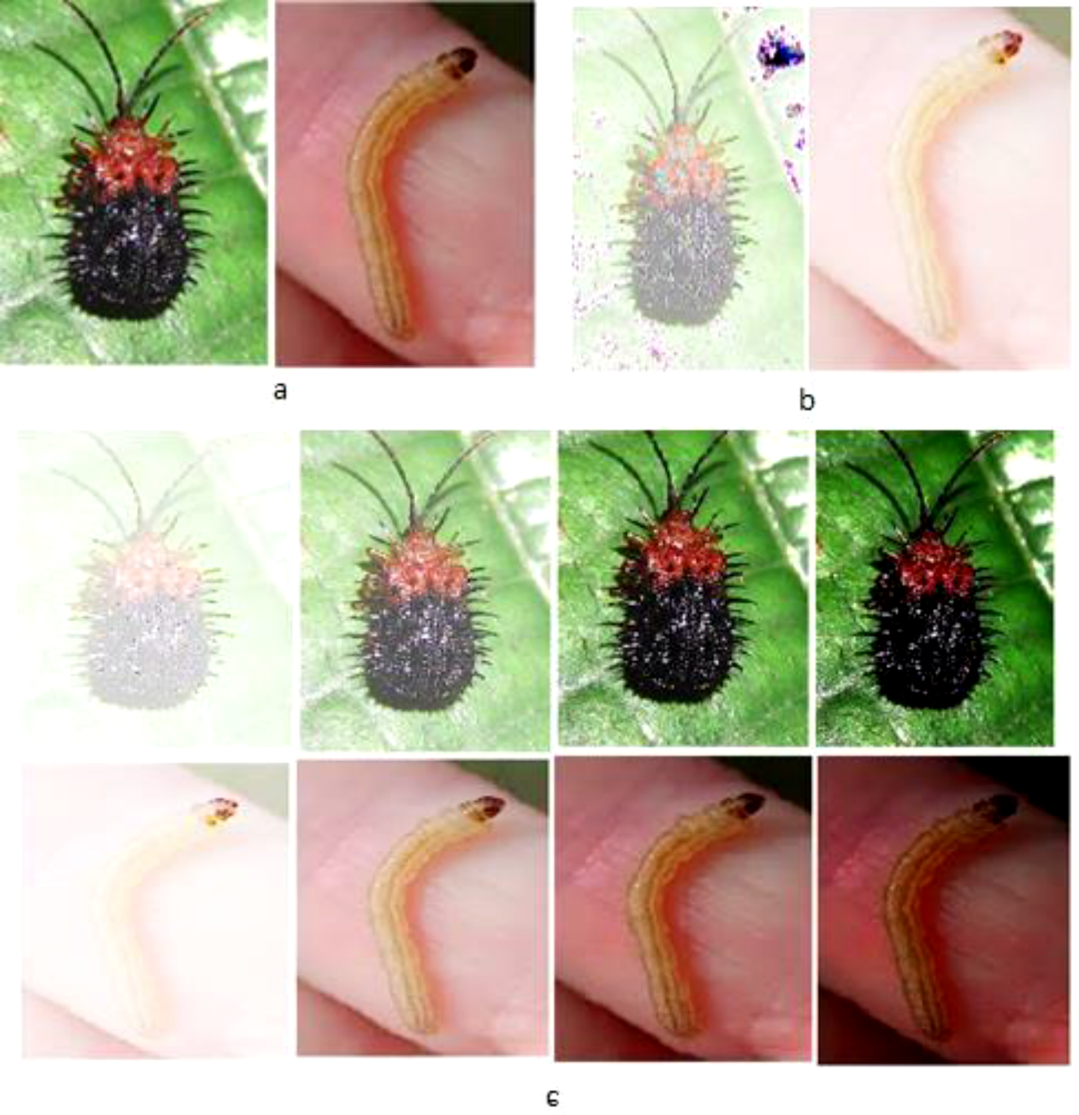
Intensity scaling techniques. (A) Original, (B) Power Law (Gamma) Transform, (C) log transform.

Hence, the application of image processing methods for leaf segmentation and removal of shadow and background focuses on the area of interest. The techniques of log transform and Power Law (Gamma) Transform, which are the sub-categorization of Intensity Scaling, were applied for background removal. Figure 7 expresses a comparative analysis of the Intensity scaling techniques. Though these methods remained deficient in the case of the utilization of the Power Low (Gamma) Transform, the complete image would be wiped off. Log transform, on another hand, controls intensity which enhances the sharpness and power of the image but can only be applied within the range of 0 to 255.

Equation (1) shows the mathematical expression of log transformation

(1) \begin{eqnarray*}\mathbi{l}=\mathbi{clog}(1+\mathbi{a})\end{eqnarray*}

l = intensity of output,

a>=0 is the pixel intensity of the input,

and c = constant of scaling.

(2) \begin{eqnarray*}\mathbi{c}=255/(\mathbf{log}(1+\mathbi{mp}))\end{eqnarray*}

where mp express as max. Pixel value which cannot be exceeded from 255, or (L-1). Log transformation maps a minimum range of low-intensity values of input to a broad range of output values.

Power-law (gamma) transformation is expressed mathematically expression in Eq. (3).

(3) \begin{eqnarray*}\mathbi{l}(\mathbi{g})=\mathbi{ca}\setminus \mathbi{gamma}\end{eqnarray*}

l = output intensity

c = scaling constant

a = input intensity of pixels.

Technique color thresholding is chosen to use for creating pixel mask values of colors of interest area. When color thresholding was applied to the images, the mask removed the background and shadow from the images, which are not needed. Eqs. (4) to xref[ref-type=disp-formula,rid=eqn-7]7 represents the mathematical expressions of the color thresholding techniques.

(4) \begin{eqnarray*}Binary:\mathbi{dst} \left( \mathbi{a},\mathbi{b} \right) = \left\{ \begin{array}{@{}l@{}} \displaystyle \mathbi{maxwalifsrc} \left( \mathbi{a},\mathbi{b} \right) \gt \mathbi{thrsh} \\ \displaystyle 0\mathbi{otherwise} \end{array} \right. \end{eqnarray*}

(5) \begin{eqnarray*}InvertedBinary:\mathbi{dst} \left( \mathbi{a},\mathbi{b} \right) = \left\{ \begin{array}{@{}l@{}} \displaystyle 0\mathbi{ifsrc} \left( \mathbi{a},\mathbi{b} \right) \gt \mathbi{thrsh} \\ \displaystyle \mathbi{maxwalotherwise} \end{array} \right. \end{eqnarray*}

(6) \begin{eqnarray*}Truncated:\mathbi{dst} \left( \mathbi{a},\mathbi{b} \right) = \left\{ \begin{array}{@{}l@{}} \displaystyle \mathbi{thresholdsrc} \left( \mathbi{a},\mathbi{b} \right) \gt \mathbi{thrsh} \\ \displaystyle \mathbi{src} \left( \mathbi{a},\mathbi{b} \right) \mathbi{otherwise} \end{array} \right. \end{eqnarray*}

(7) \begin{eqnarray*}ToZero:\mathbi{dst} \left( \mathbi{a},\mathbi{b} \right) = \left\{ \begin{array}{@{}l@{}} \displaystyle \mathbi{src} \left( \mathbi{a},\mathbi{b} \right) \mathbi{src} \left( \mathbi{a},\mathbi{b} \right) \gt \mathbi{thrsh} \\ \displaystyle 0\mathbi{otherwise} \end{array} \right. \end{eqnarray*}

(8) \begin{eqnarray*}ToZeroinverted:\mathbi{dst} \left( \mathbi{a},\mathbi{b} \right) = \left\{ \begin{array}{@{}l@{}} \displaystyle 0\mathbi{src} \left( \mathbi{a},\mathbi{b} \right) \gt \mathbi{thrsh} \\ \displaystyle \mathbi{src}(\mathbi{a},\mathbi{b})\mathbi{otherwise} \end{array} \right. \end{eqnarray*}

**Figure 8:**
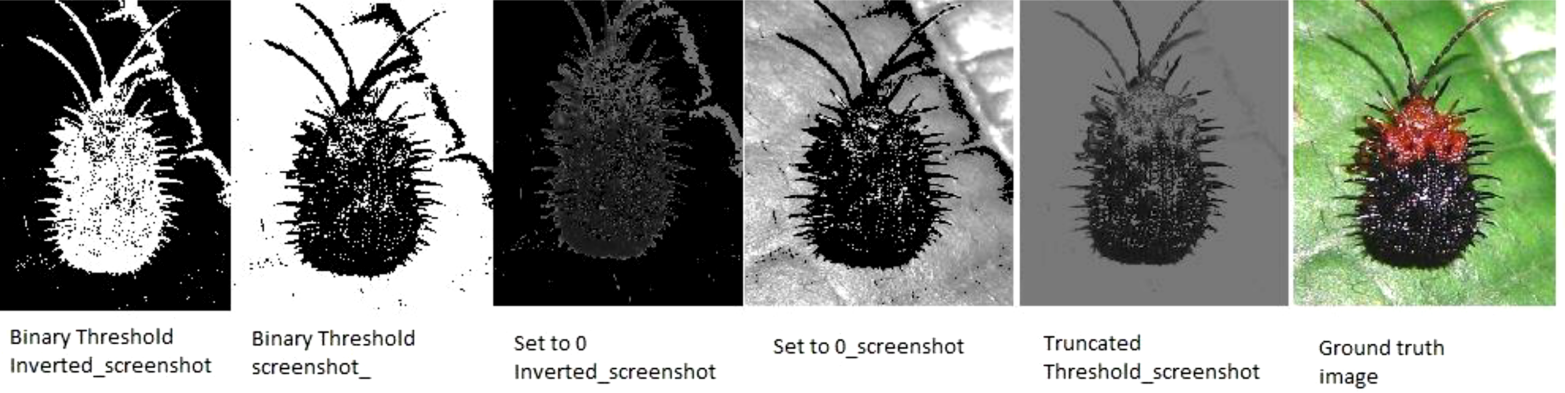
Thresholding techniques for every pixel in the images (Hispa).

**Figure 9:**
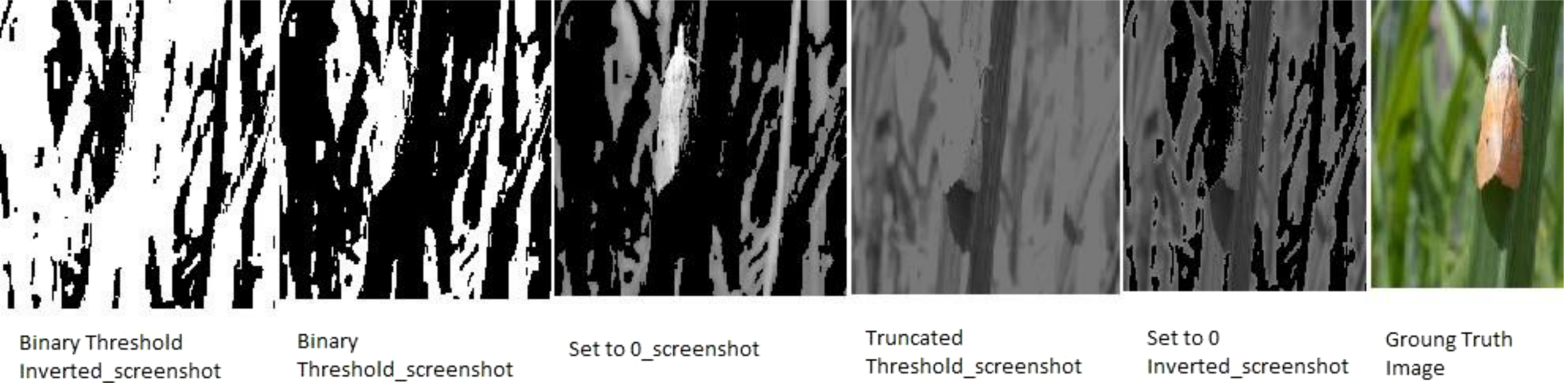
Thresholding techniques for every pixel in the images (stem borer).

Six different color thresholding techniques are applied in Figs. 8 and 9, such as binary threshold inverted, binary threshold, set to 0 inverted, set to 0, and truncated threshold. The binary threshold inverted is the opposite of binary thresholding, the destination pixel is set to zero, and if the pixel is greater than the threshold, the max value of the threshold is considered if the source pixel is less than 1. The truncated threshold is applied on the ground truth value threshold to convert the image into the pixelated format to extract the pixel value and compare it with the binary values, if it is said to be 1, then it is the minimum threshold or if the −1 then the value of the threshold is maximum. After applying and comparing the color thresholding technique, we choose the binary threshold inverted and set it to 0 technique because it gives clear images.

### Image data augmentation

**Figure 10:**
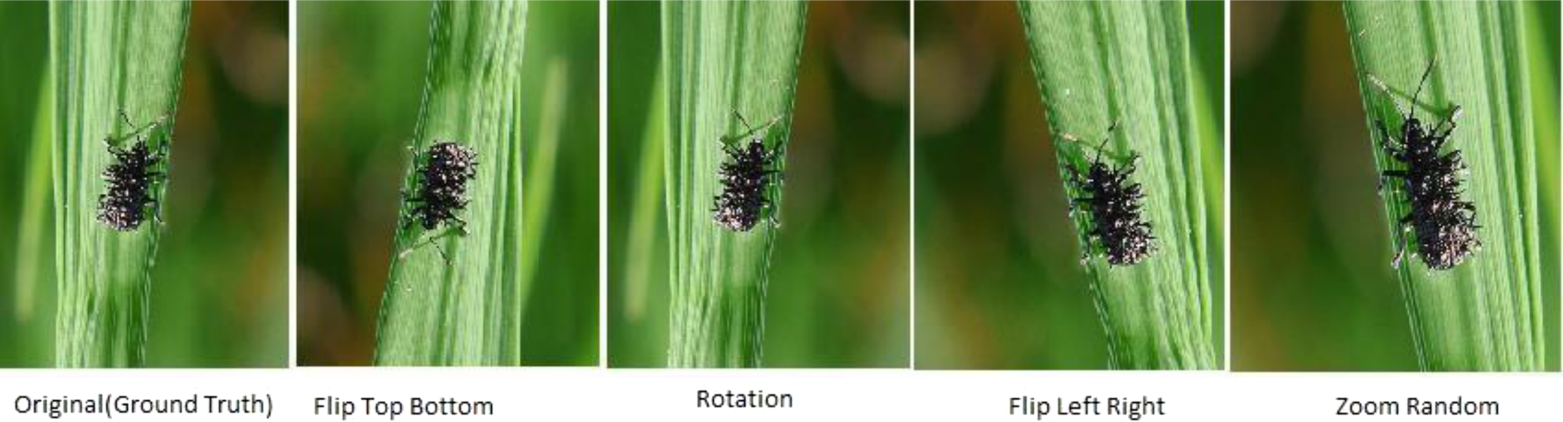
Applied augmentation techniques on images of Hispa.

**Figure 11:**
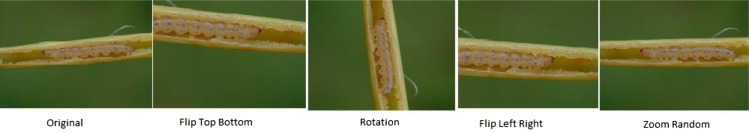
Applied augmentation techniques on images of stem borer.

The data augmentation techniques are applied to increase the data size and reduce the overfitting problem common to the data augmentation process. The augmented images are shown in Figs. 10 and 11 for training the model at different angles for precise detection and fine-tuning the parameters shown in Table 3.

**Table 3:** Fine-tuning parameters with ranges of data augmentation.

**Augmentation parameters**	**Ranges unit**
Zoom_Random	0.5
Flip_Left_Right	0.5
Rotation	10
Flip_Top_Bottom	0.5

**Figure 12:**
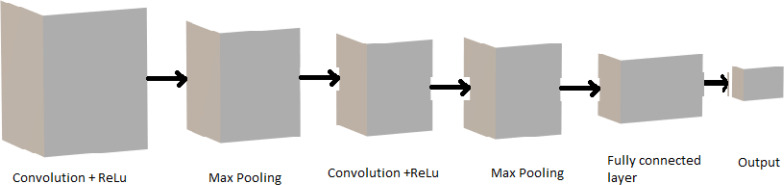
CNN based image detector architecture of the proposed backbone.

**Figure 13:**
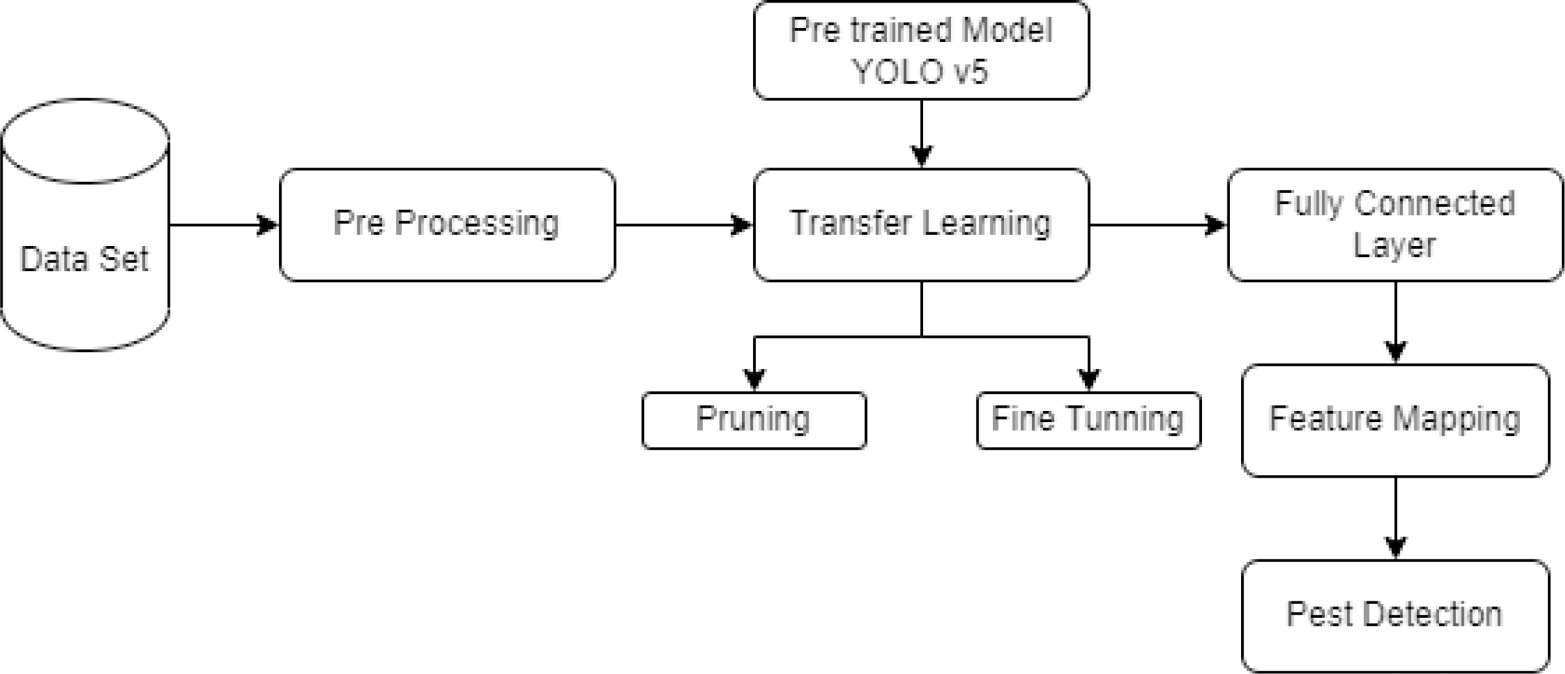
Block diagram for rice pest detection.

This section explains the four sub-sections: training and testing of data, performance matrix, comparative analysis, and the modified YO-CNN. In training and testing data, object detector models, different backbone architectures, hardware, and software specifications used for training algorithms are mentioned in detail, along with Fig. 12 and Table 4. The performance matrix Eqs (9) to (11) explains the performance measures. The comparison of different algorithms in Table 5 is provided in the comparative analysis section, which helps to identify the performance of different architectures and modify them accordingly. The custom YO-CNN explains all the measures taken to customize the model. The summary architecture and parameters of modified YO-CNN in Tables 5, 6, and Fig. 13 and its performance compared with other models are also provided.

**Table 4:** Hardware and software environments for research implementation.

**Hardware environment**		**Software environment**	
Memory	256 GB	System	ubuntu 18.0
CPU	intel® Xeon(R) Silver 4214 CPU @ 2.20 GHz ×48	Environment	Python 3.6
GPU	NVIDIA Jetson Xavier		Pytorch framework
	NVIDIA Tesla T4		

**Table 5:** Comparative analysis of models.

**Algorithm**	**Precision**
Vgg16	0.901
Vgg19	0.90
ResNet50	0.880
ResNet50v2	0.899
ResNet101v2	0.901
YOLO v5	0.910

**Table 6:** Summary of YO-CNN.

**S.no**	**From**	**n**	**Parameters**	**Module**	**Arguments**
0	−1	1	3,520	models.common.Conv	[3, 32, 6, 2, 2]
1	−1	1	18,560	models.common.Conv	[32, 64, 3, 2]
2	−1	1	18,816	models.common.C3	[64, 64, 1]
3	−1	1	73,984	models.common.Conv	[64, 128, 3, 2]
4	−1	2	115,712	models.common.C3	[128, 128, 2]
5	−1	1	295,424	models.common.Conv	[128, 256, 3, 2]
6	−1	3	625,152	models.common.C3	[256, 256, 3]
7	−1	1	1,180,672	models.common.Conv	[256, 512, 3, 2]
8	−1	1	1,182,720	models.common.C3	[512, 512, 1]
9	−1	1	656,896	models.common.SPPF	[512, 512, 5]
10	−1	1	131,584	models.common.Conv	[512, 256, 1, 1]
11	−1	1	0	torch.nn.modules.upsampling.Upsample	[None, 2, ’nearest’]
12	[−1, 6]	1	0	models.common.Concat	[1]
13	−1	1	361,984	models.common.C3	[512, 256, 1, False]
14	−1	1	33,024	models.common.Conv	[256, 128, 1, 1]
15	−1	1	0	torch.nn.modules.upsampling.Upsample	[None, 2, ’nearest’]
16	[−1, 4]	1	0	models.common.Concat	[1]
17	−1	1	90,880	models.common.C3	[256, 128, 1, False]
18	-1	1	147,712	models.common.Conv	[128, 128, 3, 2]
19	[−1, 14]	1	0	models.common.Concat	[1]
20	−1	1	296,448	models.common.C3	[256, 256, 1, False]
21	−1	1	590,336	models.common.Conv	[256, 256, 3, 2]
22	[−1, 10]	1	0	models.common.Concat	[1]
23	−1	1	1,182,720	models.common.C3	[512, 512, 1, False]
24	[17, 20, 23]	1	59,334	models.yolo.Detect	[17, [[10, 13, 16, 30, 33, 23], [30, 61, 62, 45, 59, 119], [116, 90, 156, 198, 373, 326]], [128, 256, 512]]

**Notes.**

Model Summary: 270 layers, 7,065,478 parameters, 7,065,478 gradients, 16.0 GFLOPs.

### Training and testing of data

The image or object detector is based on CNN (Das, Hazarika & Bora, 2021), an object detector that is two-stage detection of objects. In this framework, the initial stage is replaced with the other CNN, which is considered a backbone, such as Vgg16, Vgg19, ResNet50, ResNet50V2, ResNet101V2, and YOLOv5. The framework architecture is shown in Fig. 12. We propose a modified CNN architecture which is still an end-to-end detection network as a faster RCNN.

The image detector is based on CNN, which has three blocks: head, backbone, and output. The modification of the model depends on the best outcome of the stated models.

#### The CNN architecture

A convolutional neural network (CNN) is an artificial class of neural network that has become very famous in different tasks of computer vision (CV). It is also becoming dominant in other fields such as radiology. To learn spatial hierarchies of features repeatedly, adaptively, and automatically, CNN is designed perfectly for this purpose due to backpropagation using multiple building blocks (Zhang et al., 2017a).

CNN is a mathematical construct typically comprised of three building blocks or layers: convolution layer, pooling layer, and fully connected layer. The first two layers of convolution and pooling execute feature extraction, and the third, a fully connected layer, map the features extracted from the image into the final desired output, which is termed classification (Kuenzer & Knauer, 2013).

#### Models training and evaluation

The research work is evaluated by using Vgg16 and Vgg19.ResNet50, ResNet50V2, ResNet101V2, and YOLOv5 model, and on comparing these models, the custom YO-CNN is proposed. The presented work is performed using the NVIDIA JETSON AGX XAVIER controller, and we have evaluated the processing performed based on the RAM utilization, Inference Time, Temperature, and GPU utilization at different resolutions. The overall software and hardware packages required to train and test the system are shown in Table 4.

#### Performance metrics

The estimation metrics (Raghavan, Bollmann & Jung, 1989) are used to be calculated to figure out the precision of the model, so the estimation metrics of the rice pest detection model for YO-CNN are expressed in the below-stated equations.

(9) \begin{eqnarray*}\mathbi{Precision} \left( \mathbi{Pi} \right) = \frac{\mathbi{nTS}}{\mathbi{nS}} \end{eqnarray*}

(10) \begin{eqnarray*}\mathbi{Recall} \left( \mathbi{Ri} \right) = \frac{\mathbi{nTS}}{\mathbi{ntS}} \end{eqnarray*}

(11) \begin{eqnarray*}\mathbi{Avg}(\mathbi{Pi})=\sum _{\mathbi{i}=0}^{\mathbi{i}=\mathbi{n}-\mathbi{I}} \left[ \mathbi{Ra}-\mathbi{Ri} \left( \mathbi{i}+1 \right) \right] \ast Pa \end{eqnarray*}

In Eq. (9), precision evaluation is carried out in which nTS represents the number of true spots and nS represents several true spots. In Eq. (10), the recall function is calculated, including the bounded and unbounded spots. The same spots are considered once. Average precision is calculated in Eq. (11).

#### Comparative analysis

The implemented algorithms evaluation is represented in Table 5, which shows the average accuracy of Vgg16 and Vgg19.ResNet50, ResNet50V2, ResNet101V2 and YOLOv5.

This research is based on the deep learning algorithms Res Net, VGG16, and YOLO. The VGG16 shape is like a pyramid or bottleneck where the layers become deeper while moving forward and input layers are wide. VGG16 is easy to access, and pre-trained models are also available online easily, but training VGG16 requires more significant time, which exhausts GPU.

On another side, many residual modules consist of Res Net, which performs either input function or not. These modules are stacked to form the architecture of a connected network on top of one another. Res Net allows to addition or removal of the residual layers but becomes more complex and consumes time when the network gets deeper.

YOLO consists of ‘three convolutional neural network backbone layers: input layer, hidden layer, and output layer. The YOLO works well for multiple objects, with each object associated with one grid cell, which is helpful in case of overlapping where one grid cell contains the center points of the two objects known as anchor boxes. Each bounding box in the anchor box contains a certain height and width. It has fewer number layers and requires a large amount of data. High-end GPU requires processing due to big data and processing time. The misclassification also occurs if we do not have a large amount of data for each class (minimum 1k for each class) and if the data is not labeled accurately.

The comparison of the CNNs model in Table 4 shows that the winner or best performer model was YOLOv5 with a precision of 0.91 on real data.

### The proposed approach YO-CNN

The proposed approach recommended in this study is described in this section. This research starts with the input of real-time images and progresses to building a sentiment classification convolutional neural network (CNN). The network is composed of 213 layers. Every group of neurons in a layer passes through the max-pooling, reducing dimensionality to get its most excellent possible value as a contribution. “A new deep learning-based technique for rice pest detection using remote sensing” is achieved in the shortest period than existing techniques (Mahle, 2016; Pethybridge & Nelson, 2015; Stewart & McDonald, 2014; Bock et al., 2009; Bai et al., 2017; Zhang et al., 2017a; Dorj, Lee & Yun, 2017; Zhang et al., 2017b; Du et al., 2016; Atole, 2018). The network receives a preprocessed 64 x 64 pixel as input. Using the results of the previous model, a new model was developed as YO-CNN. A smaller network was regarded to be better than a bigger one. Using additional layers has the advantage of preventing memorization. A broad but shallow network is good at memorizing but not at generalizing. It is possible to generalize successfully using multi-layer networks since they can learn properties at multiple abstraction levels. For practical uses, the 213 layers were selected to assure a high level of precision. Four convolutional layers, each with a distinct filter size, are included in the proposed CNN, distinguishing it from a basic CNN. To further prevent overfitting, it utilized max-pooling and dropouts more effectively.

The YO-CNN accomplishes more detection results than Vgg16, Vgg19, ResNet50, ResNet50V2, ResNet101V2, and YOLOv5. The architecture YO-CNN reduces the model size and increases the precision. Figure 13 shows the working block diagram, including YO-CNN, which detects rice pests.

The YO-CNN is used because it is a modified, improved, and optimized algorithm designed to reduce the size of the image and tuning of hyperparameters. The existing algorithms are not optimized enough to work on image classification precisely to promote a smart agriculture system.

A detailed description of YO-CNN is provided in Table 6, in which modules are the number of models having 24 convolution layers and 270 overall layers, including max pooling, fully connected layers, convolution layers, and present Adams as an optimization layer. The total number of parameters is 7,065,478, representing the trained model’s hyperparameters.

The model is trained with CNN based technique which has three parts:

#### Backbone: CSP darknet

The initial part of the YO-CNN represents the cross-stage partial network into the darknet, which creates the CSP darknet as its backbone. It solves the gradient information, which is repeated in the backbone. The gradient integrates the gradients by changing the feature map; therefore, decreasing the FLOPS and parameters of the model will guarantee the accuracy, model, and speed of the inferences. In the rice pest detection task, the detection speed and accuracy are imperative, and the model size is smaller than others.

#### Neck: PA net

In the second stage, data is aggregated for passing the boost information flow, which links the feature grid and feature levels.

#### Output layer

The third layer output layer acts as a binary classifier, which is responsible for learning the entire picture to identify the images that contain pests’ objects. Finally, the pest detection and classification results are directed into a decision-making module, which detects pests like HISPA and STEM BORER from images.

### The algorithm of YO-CNN

The pseudo-code in Table 7 is provided below, which describes the entire procedure of the modified YO-CNN model.

**Table 7:** Pseudo-code for explaining the work flow of model.

**Algorithm YO-CNN**
**Input:** Rice Pest images with annotation files.
**Output:** Trained Rice Pest Detection (YO-CNN) Model
**Processing Steps:**
1. If a set is training data set, then follow steps 2 to 4.
2. pre-processing to resize the image (640 × 640)
3. normalize pixel values [0, 1]
4. standardize pixel values to (640 × 640)
5. augment the data with different augmentation techniques
6. Apply Upsampling technique to reshape the shapes of input parameters
7. Model training with MODEL = YOLO v5
8. Set epoch = 0 to 100.
9. Set learning rate as Lr= 0.01 use steps 13 to 14
10. Set g0 as optimizer parameter group
11. for a model selection use steps 7 to 10
12. If OPTIMIZER == Adam: optimizer = Adam (g0, Lr=hyp[‘Lr0’], betas= (hyp[‘momentum’], 0.937)) else optimizer = SGD (g0, Lr=hyp[‘Lr0’], betas= (hyp[‘momentum’], nesterov=True))
13. In a batch of no of images: 456
14. update model parameter
15. end of for loop of step 14
16. Training of the model parameters started
17. End of training step 16
18. for testing no of images in batch: update model parameter
19. end of for loop of step 18

The accuracy of proposed model YO-CNN is 0.980, which is more precise than existing models, as mentioned in Table 8.

**Table 8:** Training parameters and accuracy of models in comparison with YO-CNN.

**Training parameters and accuracy**							
Model	Labels	Image size	Batch size	Weight size	Recall	Precision	mAP
YO-CNN	2	640 ×640	16	14.5 MB	0.976	0.97	0.98
Vgg16				528	0.88	0.901	0.923
Vgg19				549	0.89	0.900	0.921
ResNet50				98	0.879	0.88	0.890
ResNet50v2				100	0.889	0.899	0.90
ResNet101v2				109	0.89	0.901	0.91
YOLOv5				14MB	0.90	0.91	0.91

## Results

**Figure 14:**
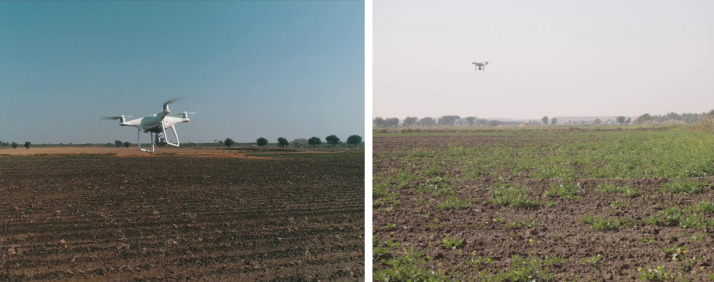
Drone while collecting rice pests’ data.

**Figure 15:**
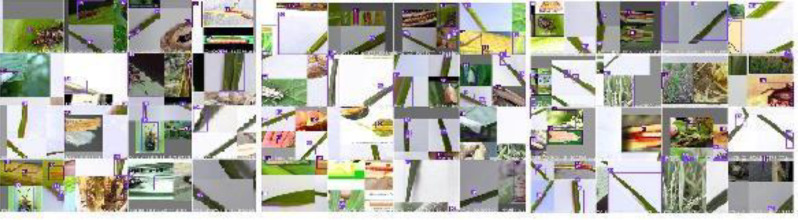
YO-CNN detection of pests.

**Figure 16:**
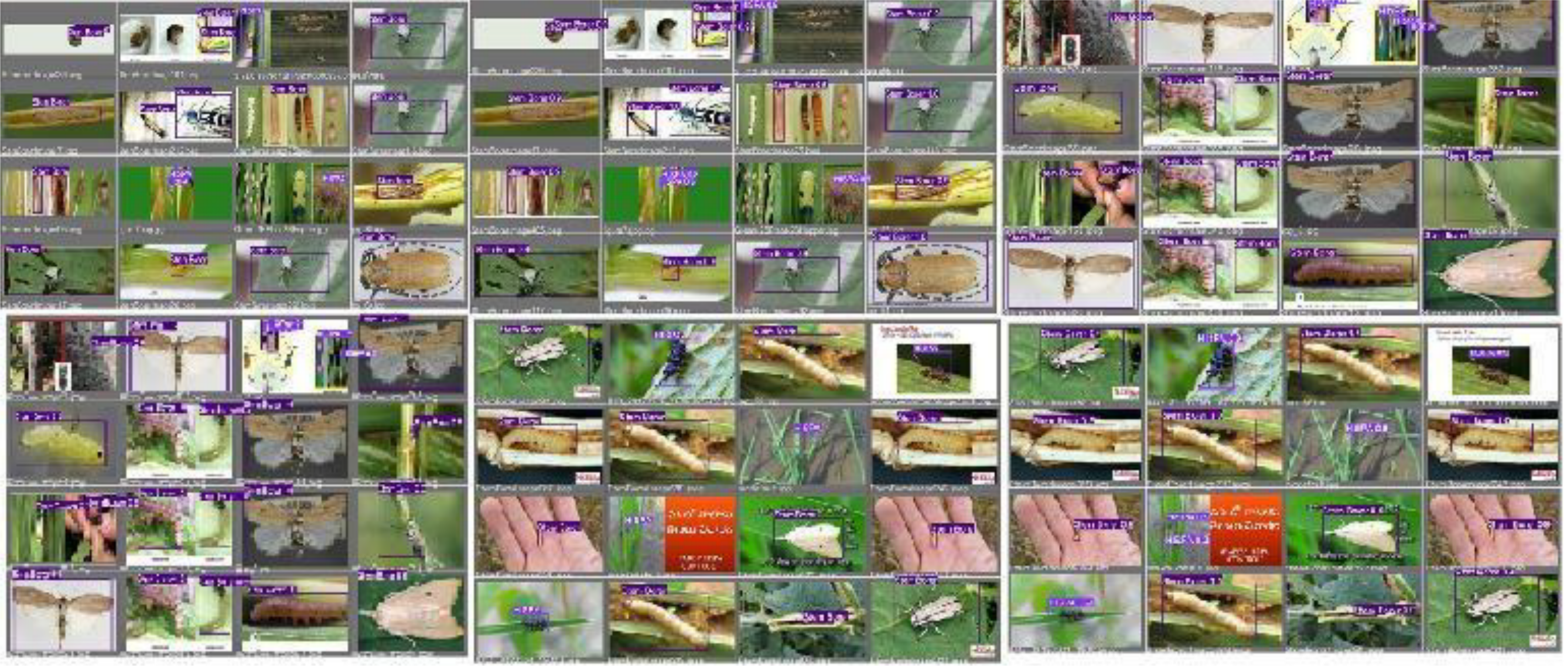
YO-CNN validation of pests.

**Figure 17:**
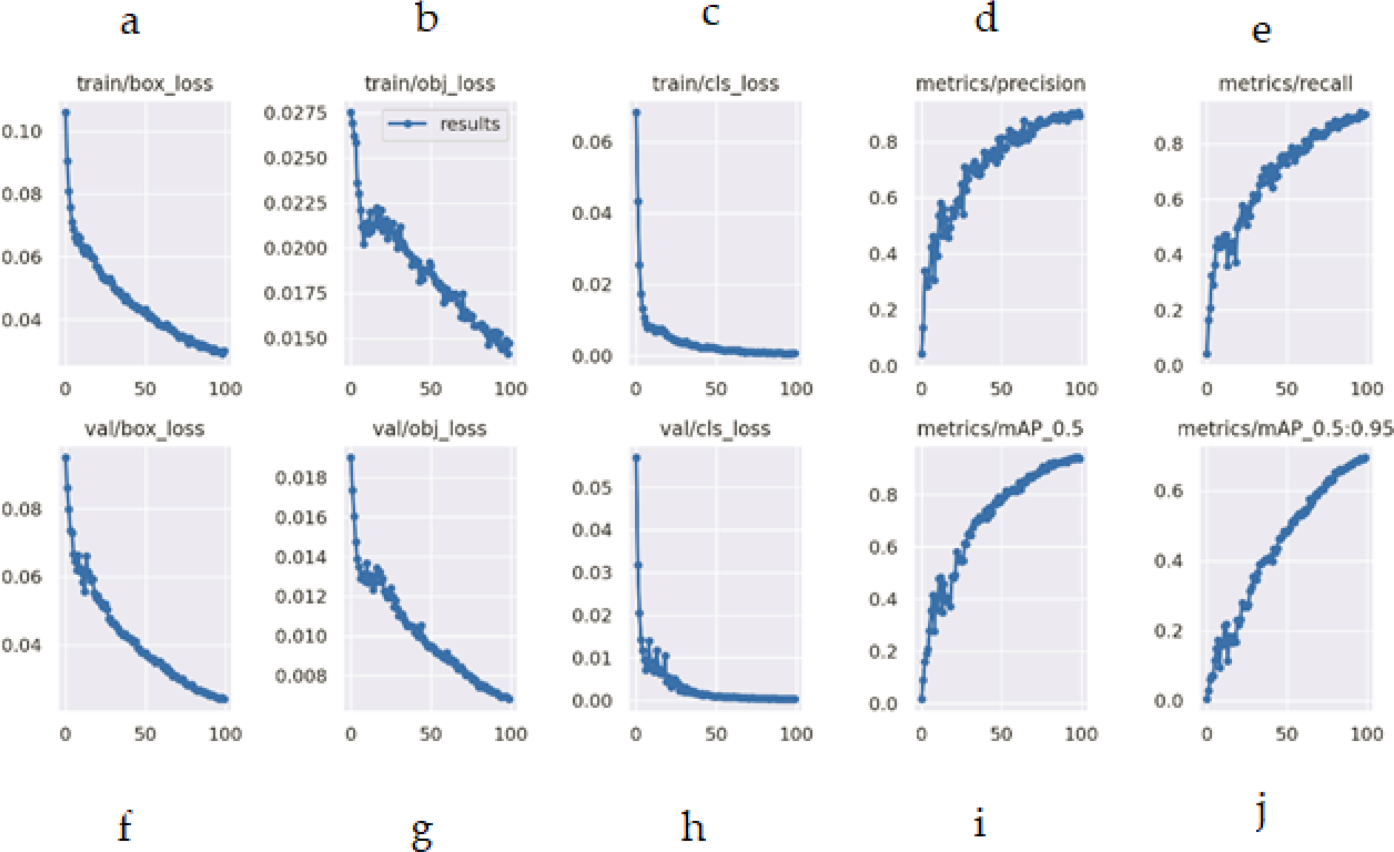
(A–J) Evaluation metrics of YO-CNN.

**Figure 18:**
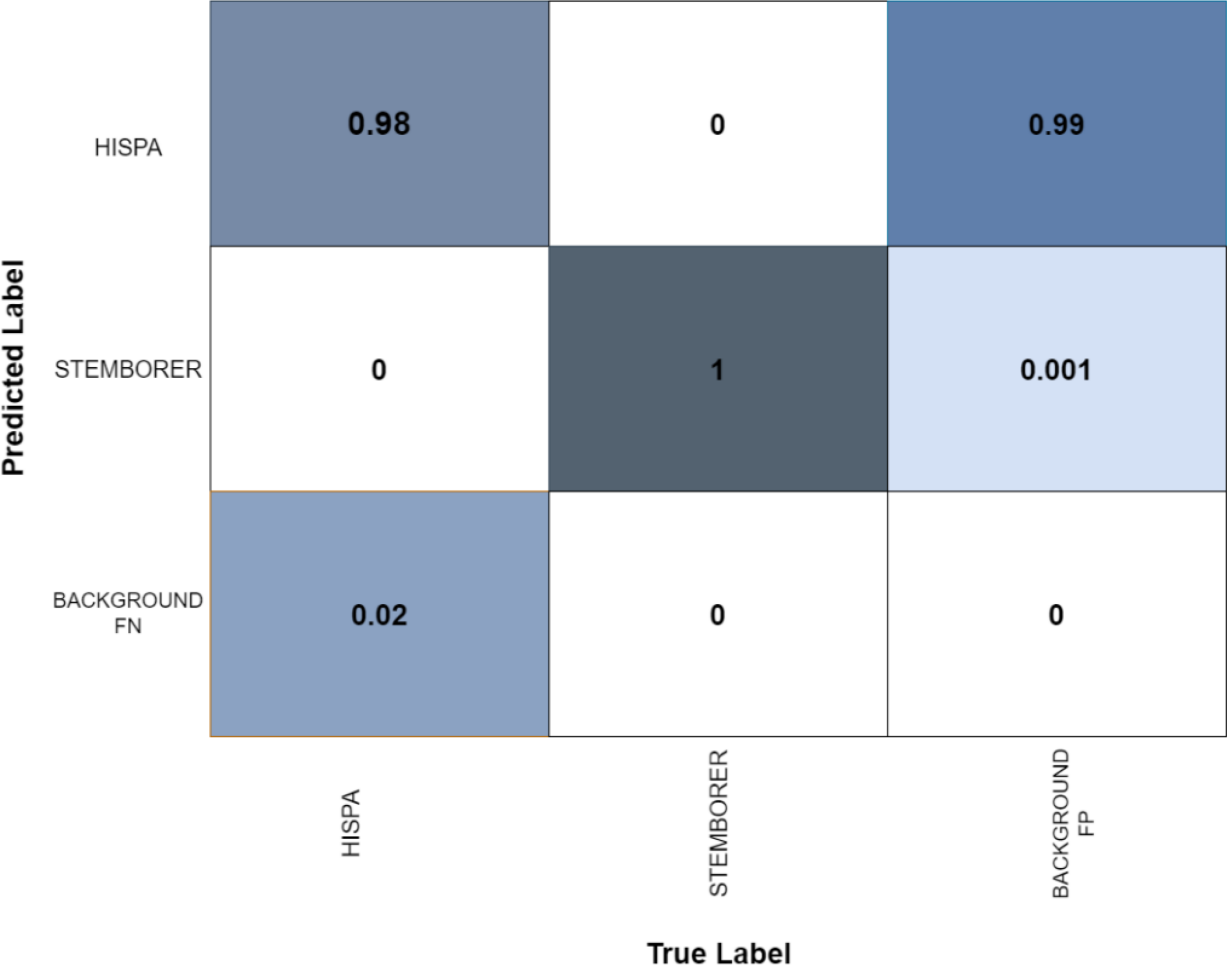
Confusion matrix of stem borer and Hispa.

This section composes of two more subsections: the Results of the pests detection and the confusion matrix. In the subsection, results of the pest’s detection, the original images are provided, which are obtained during training, processing, testing, evaluation, and validation which may visualize in Figs. 14, 15, 16 and 17. The confusion matrix provides the confusion analysis in Fig. 18.

### Results of the pest detection

In Fig. 14, the UAV is used for monitoring the rice field so that the detection of pests can be done at this early stage. It helps the farmers to do operations for controlling pests which results in increasing yield, and also safe pesticide sprays harmful to the environment and living beings.

The results of image detection during training are shown in Fig. 15. Light purple with the number 15 shows Hispa, and dark purple boxes with the number 16 show stem borer. The two categories of rice pests, stem borer, and Hispa, can be spotted concurrently. The touching boundaries may be the reflection of dissimilar grazes. The detection speed was about 0.025s due to the high processing unit.

In Fig. 16, validation of pests can be visualized as the name of classified pests stem borer or Hispa are written along with bounding boxes.

Figure 17 shows the losses, precision, recall, and average precision. Losses can be visualized during training and validation on the left side of the figure. All the losses are falling from the maximum position while the graph of precisions is increasing, and the average accuracy of custom YO-CNN is about 0.980.

### Confusion matrix

The confusion matrix is provided in Fig. 18, which shows the complete picture of confusion faced by the detector as the detector would be confused in Hispa and stem borer. The confusion may occur due to blur or the complex background of images. In the matrix, it is clearly shown that the accuracy of the model misses very few points. Figure 18 shows the confusion matrix below.

## Discussion

**Figure 19:**
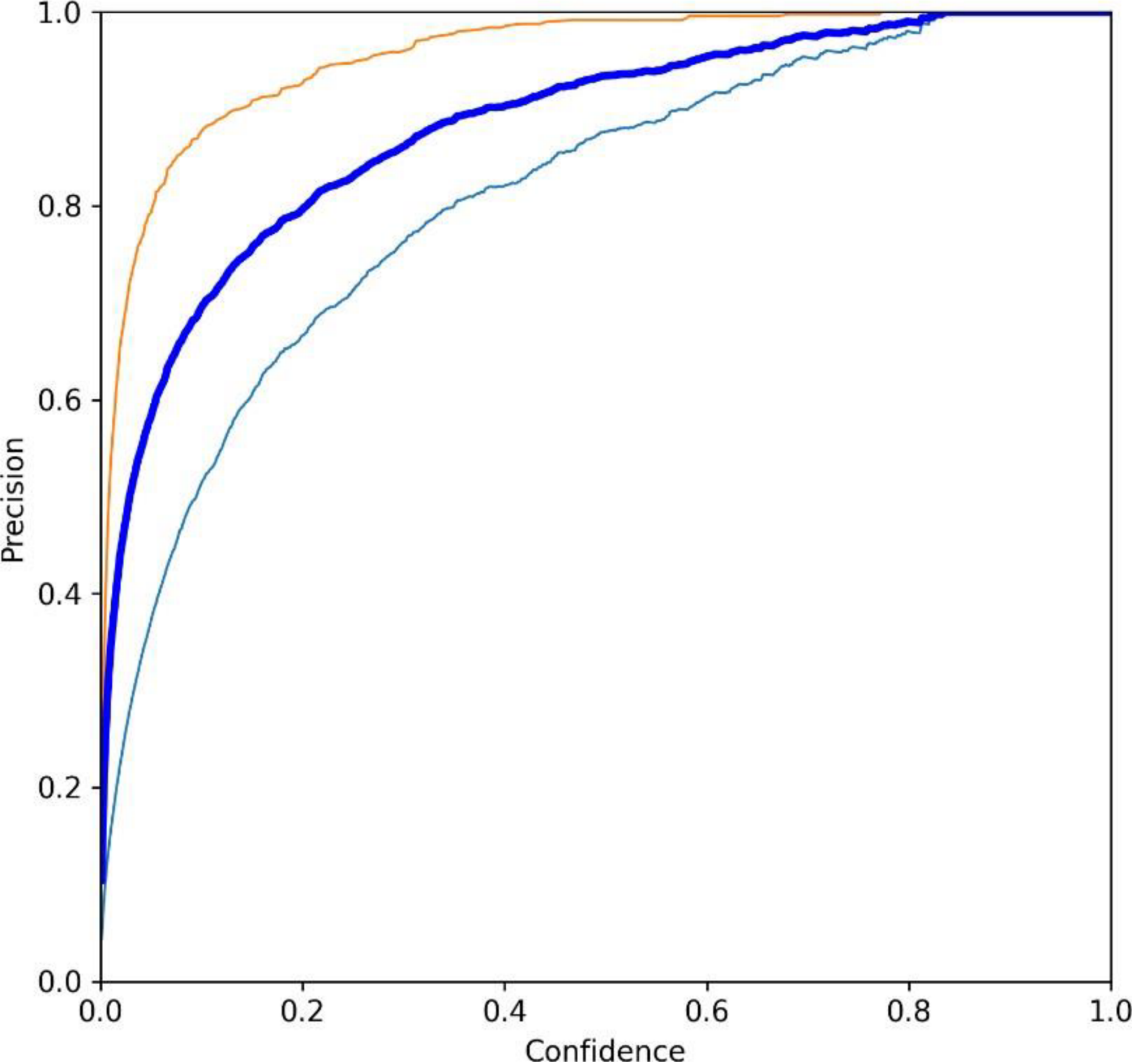
Precision curve of YO-CNN.

**Figure 20:**
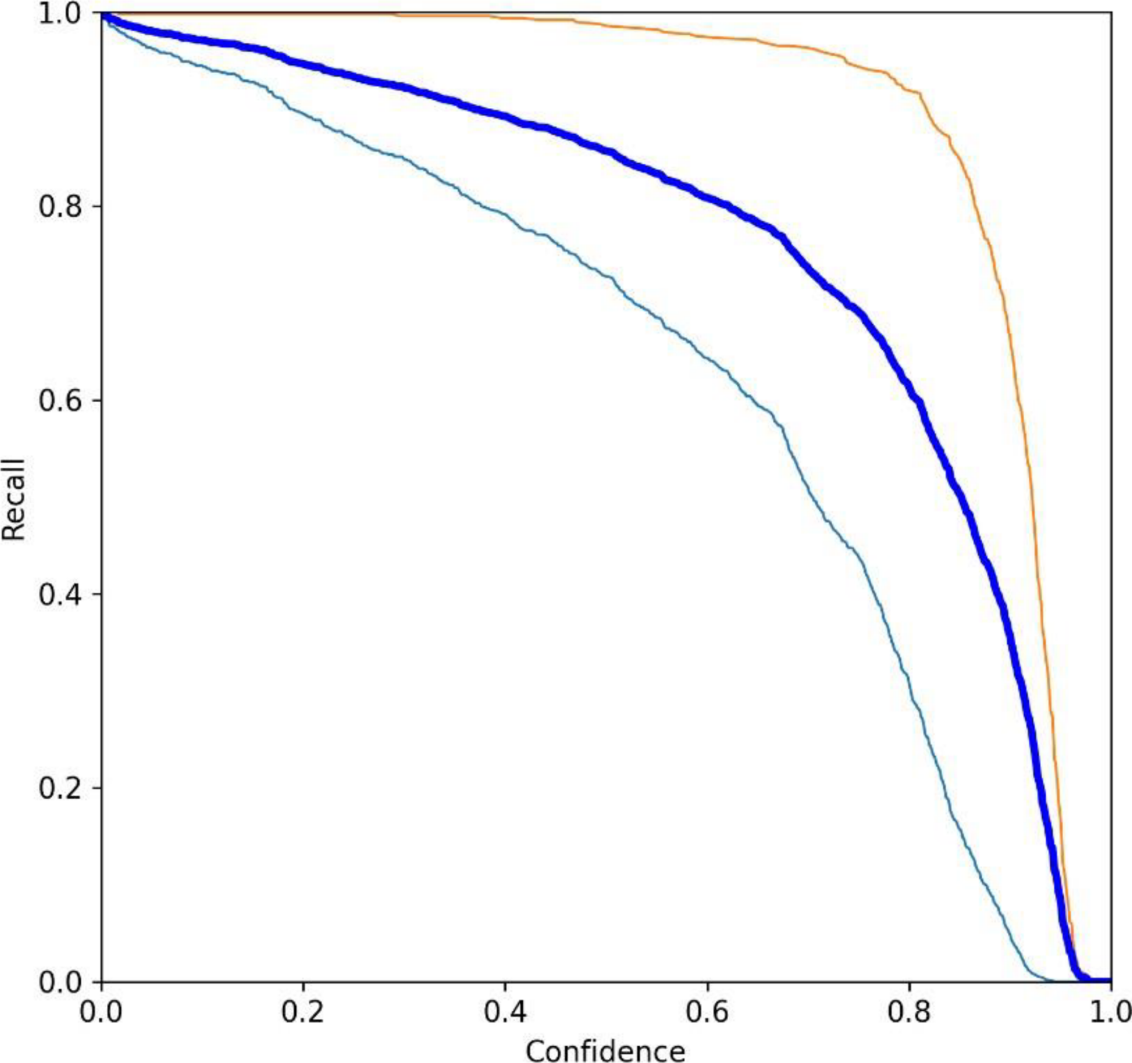
Recall curve of YO-CNN.

This section provides a comparative analysis of existing techniques, showing differences in the customized model YO-CNN achievement. Table 9 is designed to show the comparison of YO-CNN with existing technologies. The outcome of YO-CNN is more precise, which is the standout feature of this research. Moreover, it can be utilized in other agriculture scenarios with other data sets related to the application. Figures 19 and 20 show the precision and recall curve.

**Table 9:** Average accuracy of algorithm in comparison with customized YO-CNN.

**Algorithm**	**Average accuracy**
YO-CNN	0.980
Vgg16	0.923
Vgg19	0.921
ResNet50	0.890
ResNet50v2	0.90
ResNet101v2	0.910
YOLO v5	0.910

### Comparative analysis

The comparison of YO-CNN with other models VGG16, VGG19 ResNet-50, ResNet50v2, ResNet-101v2, and YOLOv5, is provided in Table 8. Their precision shows the efficiency of models and declares that the modified YO-CNN is much better than the existing models. The current models VGG16, VGG19 ResNet-50, ResNet50v2, ResNet-101v2, and YOLOv5 might have an unacceptable finding result on a little blur effect on images while YO-CNN reduces this problem too as verification of the YO-CNN on Hispa and stem borer finds that YO-CNN performs better for object detection with blur boundaries and uneven surfaces. The average accuracy of the custom YO-CNN is better than other architectures.

Figures 19 and 20 show the precision curve and recall curve for the Hispa and Stem borer, which offers the performance of YO-CNN. The curve with light blue color represents Hispa, Curve with red color represents Stem borer, and the curve with dark blue color, which is in between these two curves, represents the cumulative performance of both classes.

## Conclusions

Identifying the pests at the right time is essential for protecting and increasing the yield, which helps in growing healthy crops and minimizing the use of pest spray, increasing yield, and reducing human effort. In this article, the image detection of two categories of rice pests, Hispa and stem borer, using the new transfer learning-based model YO-CNN is successfully achieved. Features are extracted using fully connected layers. The image detection is performed with different algorithms and provides their comparative analysis in Table 5 shows YOLO v5 is more accurate than 0.910 accuracies. We modified YOLO v5 and provided a detailed summary of customizing YO-CNN in Table 6 and pseudo-code in Table 7. A design of modified YO-CNN architecture for pest detection is presented, which is more suitable for pest of rice image detection with the accuracy of 0.980 shown in Table 9. We notice that VGG16, VGG19 ResNet-50, ResNet50v2, ResNet-101v2, and YOLOv5 might have an unacceptable finding result on a little blur effect on images. Also, we verify the YO-CNN on Hispa and stem borer and find that YO-CNN performs better with an accuracy of 0.980 for object detection with blur boundaries and uneven surfaces and shapes compared with other algorithms, which is a standout feature of this result. The proposed system can be applied to other crops for pre-detection after suitable modification. The future of this finding is to adopt this method for precise spraying with an unmanned aerial vehicle which helps to spray on the targeted location in an adequate amount and precisely to optimize the resources, human effort, and harmful effects on nature.

##  Supplemental Information

10.7717/peerj-cs.1167/supp-1 Supplemental Information 1 Image Augmentation coding for increasing the data set and to have images in different angles to train the model efficiently

10.7717/peerj-cs.1167/supp-2 Supplemental Information 2 Programming for clearing the features of the image to select and identify the region of interest more easily pre processing

10.7717/peerj-cs.1167/supp-3 Supplemental Information 3 Coding of training and validation of a model to detect, Identify and classify the pest stress areas

## Additional Information and Declarations

### Data Availability

The following information was supplied regarding data availability: The data is available at Figshare: Hassan, Syeda (2022): Rice Pest D1. figshare. Figure. https://doi.org/10.6084/m9.figshare.19918981.v1
Hassan, Syeda (2022): Rice Pest D2. figshare. Figure. https://doi.org/10.6084/m9.figshare.19918987.v1
Hassan, Syeda (2022): Rice Pest D3. figshare. Figure. https://doi.org/10.6084/m9.figshare.21695411.v1
Hassan, Syeda (2022): Rice Pest D4. figshare. Figure. https://doi.org/10.6084/m9.figshare.19918999.v1
Hassan, Syeda (2022): Rice Pest D5. figshare. Figure. https://doi.org/10.6084/m9.figshare.19919020.v1.
