# Supplementary material for: A new deep learning-based technique for rice pest detection using remote sensing
Source: PeerJ Comput Sci. 2023 Mar 6;9:e1167. doi: 10.7717/peerj-cs.1167 (PMC10280224; doi:10.7717/peerj-cs.1167)
Supplement: Supplemental Information 3 [file peerj-cs-09-1167-s003.docx]

**Algorithm YO-CNN:**

Input: Rice Pest images with annotation files.

Output: Trained Rice Pest Detection (YO-CNN) Model

Processing Steps:

1. If a set is training data set, then follow steps 2 to 4.

2. pre-processing to resize the image (640 × 640)

3. normalize pixel values [0, 1]

4. standardize pixel values to (640 × 640)

5. augment the data with different augmentation techniques

6. Apply Upsampling technique to reshape the shapes of input parameters

7. Model training with MODEL = YOLO v5

8. Set epoch = 0 to 100.

9. Set learning rate as Lr= 0.01 use steps 13 to 14

10. Set g0 as optimizer parameter group

11. for a model selection use steps 7 to 10

12. If OPTIMIZER == Adam:

optimizer = Adam (g0, Lr=hyp[‘Lr0’], betas= (hyp[‘momentum’], 0.937))

else optimizer = SGD (g0, Lr=hyp[‘Lr0’], betas= (hyp[‘momentum’], nesterov=True))

13. In a batch of no of images: 456

14. update model parameter

15. end of for loop of step 14

16. Training of the model parameters started

17. End of training step 16

18. for testing no of images in batch: update model parameter

19. end of for loop of step 18
